# Inhibition of P-glycoprotein Gene Expression and Function Enhances Triptolide-induced Hepatotoxicity in Mice

**DOI:** 10.1038/srep11747

**Published:** 2015-07-02

**Authors:** Ling-Lei Kong, Xiao-Mei zhuang, Hai-Ying Yang, Mei Yuan, Liang Xu, Hua Li

**Affiliations:** 1 State Key Laboratory of Toxicology and Medical Countermeasures, Beijing 100850, China; 2 Beijing Institute of Pharmacology and Toxicology, Beijing 100850, China

## Abstract

Triptolide (TP) is the major active principle of *Tripterygium wilfordii* Hook f. and very effective in treatment of autoimmune diseases. However, TP induced hepatotoxicity limited its clinical applications. Our previous study found that TP was a substrate of P-glycoprotein and its hepatobiliary clearance was markedly affected by P-gp modulation in sandwich-cultured rat hepatocytes. In this study, small interfering RNA (siRNA) and specific inhibitor tariquidar were used to investigate the impact of P-gp down regulation on TP-induced hepatotoxicity. The results showed that when the function of P-gp was inhibited by mdr1a-1 siRNA or tariquidar, the systemic and hepatic exposures of TP were significantly increased. The aggravated hepatotoxicity was evidenced with the remarkably lifted levels of serum biomarkers (ALT and AST) and pathological changes in liver. The other toxicological indicators (MDA, SOD and Bcl-2/Bax) were also significantly changed by P-gp inhibition. The data analysis showed that the increase of TP exposure in mice was quantitatively correlated to the enhanced hepatotoxicity, and the hepatic exposure was more relevant to the toxicity. P-gp mediated clearance played a significant role in TP detoxification. The risk of herb-drug interaction likely occurs when TP is concomitant with P-gp inhibitors or substrates in clinic.

*Tripterygium wilfordii* Hook f. (TWHF) has a long history of use in traditional Chinese medicine for the treatment of autoimmune diseases, such as nephritis, lupus erythematosus and rheumatoid arthritis^1^. Triptolide (TP) is the major active ingredient of TWHF and belongs to diterpenoid triepoxide. It has demonstrated multiple pharmacological activities, such as immunosuppressive activities, anti-inflammatory, anti-cancer, anti-fertility and neuroprotection^2^,^3^,^4^. However, relatively high rate of adverse effects has limited its clinical application. Among the adverse events of TP, high incidence of hepatotoxicity in clinical was considered as a main cause of death and has drawn great attention of physicians and toxicologists^5^,^6^,^7^,^8^.

Metabolism and pharmacokinetic studies of TP indicated that the compound could be concentrated in liver with the level significantly higher than that in other tissues^9^,^10^. TP underwent extensive metabolism to form several oxidative metabolites^11^,^12^. Cytochrome P450 3 A (CYP3A) was found to be the major enzyme responsible for TP metabolism^13^,^14^. *An in vitro* study using sandwich-cultured rat hepatocyte (SCRH) model showed that induction and inhibition of CYP3A could significantly alter the TP intracellular concentrations and subsequently hepatotoxicity^15^. Inactivation of hepatic CYPs in CYP reductase knockout (KO) mice resulted in the remarkably increased TP systemic exposure and toxicity^11^. Similarly, pretreatment of animals with CYP3A inhibitors or inducers could significantly alter TP-mediated hepatotoxicity by affecting its metabolic profile^12^,^16^. These studies concluded that CYP3A mediated hepatic metabolism was a major clearance and detoxification pathway of TP.

In addition to the CYP mediated metabolism, recent studies found that biliary excretion was also an important clearance route of TP. In bile duct-cannulated (BDC) rats, a nearly 39% of TP dose was excreted via bile in 24 h^17^. By using ATPase assay with rat mdr1 membrane, TP was identified as a substrate of P-glycoprotein (P-gp)^18^. P-gp is an efflux transporter that mediates the ATP-dependent efflux of drugs from cells. The P-gp protein is expressed in the apical membranes of excretory cells such as hepatocytes and plays a part in the biliary excretion of drugs. The expression level of P-gp as well as its function can be modulated by inhibition and induction, which subsequently affect the pharmacokinetics, efficacy, safety or tissue exposure of P-gp substrates^19^,^20^. Our previous study demonstrated the involvement of P-gp in the TP biliary excretion in *in vitro*^18^. Co-incubation of TP with P-gp inhibitors in SCRH could significantly enhance TP intracellular exposure and led to aggravated hepatotoxicity. The results suggested that P-gp mediated biliary efflux was also an important detoxification pathway for TP and modulating efflux transporter mediated TP clearance might cause drug-drug interaction (DDI) related safety concerns. However, the role of P-gp in TP *in vivo* clearance and detoxification remains unclear. Further investigation using animal models is necessary to evaluate the toxicological outcome of TP by P-gp modulation and to assess the potential DDI risk associated with the efflux transporter.

Interplay between CYP and P-gp is a widely reported phenomenon^21^,^22^,^23^,^24^. A considerable overlap was observed in the substrate specificity and inhibitors/inducers, such as ritonavir, ketoconazole and verapamil^25^. It is difficult to use these double functional chemicals to differentiate roles of P-gp and CYP in drug clearance and DDI. In the present study, RNA interference (RNAi) technique was used to assess the contribution of P-gp in TP clearance and toxicity in mice. RNAi is a process that causes gene knockdown in a sequence-specific manner. High specificity and efficiency of RNAi makes it a preferred tool for functional genomics research and gene therapy^26^. The technique has also been applied in drug transporter related disposition studies^27^,^28^,^29^. In the current study, a chemically synthesized small interfering RNA (siRNA) was used to specifically knock down P-gp expression in mice. The plasma and hepatic exposures of TP were determined to demonstrate the effect of P-gp knockdown on TP clearance and toxicity. Serum biochemistry and histopathology were used to evaluate the TP-induced liver injury. And the levels of malondialdehyde (MDA), superoxide dismutase (SOD) and apoptosis-related protein (Bcl-2 and Bax) expression were also measured as additional toxicological end points for analysis of toxicological mechanism of TP. Tariquidar, a specific P-gp inhibitor, was also tested in parallel with siRNA for comparison and for assessment of the efflux transporter associated DDI.

## Materials and Methods

### Chemicals and reagents

**Figure 1:**
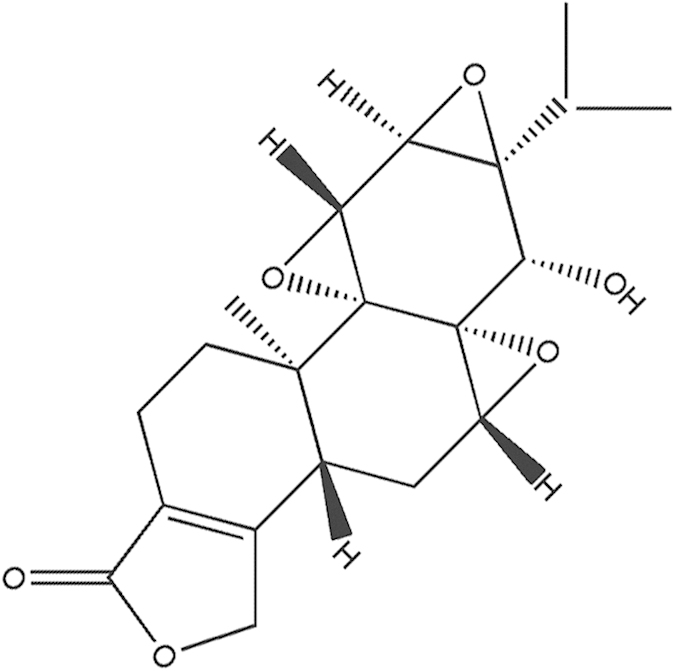
Chemical structure of triptolide.

TP (Fig. 1) was obtained from National Institutes for Food and Drug Control (Beijing, China) with the purity >99%. Tariquidar was purchased from Sigma (St. Louis, MO, USA). The siRNA was chemically synthesized by Ribobio Co., Ltd (Guangzhou, China). The rabbit anti-P-gp was obtained from Abcam (Cambridge, UK). The mouse anti-Bcl-2 and anti-Bax were purchased from Santa Cruz Biotechnology (Santa Cruz, CA, USA). Horseradish peroxidase (HRP)-conjugated secondary antibodies were from Pierce Biotechnology (Thermo Fisher Scientific, Rockford, IL, USA). Acetonitrile and Methanol were of HPLC grade (J&K Chemical LTD) and other reagents were all of analytical grade.

### Animals

Male BALB/C mice (weight, 18–22 g) were supplied by Beijing Experimental Animal Center (Beijing China). All animals were maintained on a 12-h light/dark cycle with free access to water and lab chow. All of the procedures were carried out in accordance with the standards established in the *Guide for the Care and Use of Laboratory Animals* published by the Institute of Laboratory Animal Resources of the National Research Council (United States), and also approved by the Animal Care and Use Committee of the Beijing Institute of Pharmacology and Toxicology. All the efforts were made to minimize the number of animals used and their suffering.

### Design of siRNAs

Three sequences for murine mdr1a siRNA were chosen according to Matsui *et al.*^30^ and Patutina *et al.*^31^. Their sequences were given as follows: mdr1a-1 siRNA, sense 5′-r(AAU GUU GUC UGG ACA AGCACU) d(TT)-3′ and antisense 5′-r(AGU GCU UGU CCAGAC AAC AUU) d(TT)-3; mdr1a-2 sense 5′-r(AGA AGGAAC UAG AAG GUU CUG) d(TT)-3′ and antisense 5′-r(CAG AAC CUU CUA GUU CCU UCU) d(TT)-3′; mdr1a-3 siRNA, sense 5′-GGCUGGACAAGCUGUGCAUGG-3′ and antisense 5′-AUGCACAGCUUGUCCAGCCAA-3′; negative control (NC) siRNA, sense 5′-UUCUCCGAACGUGUCACGUTT-3′ and antisense 5′-ACGUGACACGUUCGGAGAATT-3′. The 3′- and 5′- end were chemically modified with methylation and cholesterol to elevate the stability.

### Screening and functional evaluation of mdr1a siRNA *in vitro* and *in vivo*

CT26.WT cells, a mouse colon carcinoma cell line, were obtained from ATCC and maintained in RPMI-1640 supplemented with 10% fetal bovine serum. Cells were seeded to 6-well plates at a density of 5 × 10^5^ cells/well and cultured for 24 h. Transfection was performed in Opti-MEM (Invitrogen, Carlsbad, CA, USA) with synthetic siRNA (80 pM) by using Lipofectamin 2000 (Invitrogen, Carlsbad, CA, USA) for 6 h and the medium was replaced with fresh RPMI-1640. This condition was optimized in our preliminary experiments based on the transfection efficiency and the cellular viability. Following incubation for 48 h, the cells were washed three times with phosphate buffered saline buffer (PBS) and total RNA and protein were collected.

For validation of silence efficiency *in vivo*, mdr1a-1 siRNA was injected via the tail vein of mice after diluted to 5, 10 and 15 nmol with saline in 200 μl. After 48 h, the mice were euthanized and the liver was collected for measurement of the P-gp protein expression using western blot. The P-gp function in gene knockdown mice was assessed using digoxin, a known P-gp substrate. NC-siRNA or mdr1a-1 siRNA was intravenously injected 2 days prior to the injection of digoxin (1mg/kg). Blood samples were collected at 5, 15, 30, min and 1, 2, 4, 6, 8, 12 hours post digoxin dose (n = 5/time-point for each group). The digoxin concentrations in samples were quantitatively analyzed by a published LC-MS/MS method^32^.

### RNA extraction, reverse transcription (RT), and real-time PCR

Total RNA from CT26. WT cells was extracted with Trizol (Invitrogen, Carlsbad, CA, USA) according to the manufacturer’s instructions. RNA samples (1 μg) were reverse transcribed by adding oligo(dT)18 (MBI Fermentas, USA), dNTP, RNase inhibitor, and M-MLV reverse transcriptase (Takara, Japan), holding at room temperature for 10 min and incubating at 42 °C for 60 min. The samples were heated at 72 °C to inactivate enzymes for 10 min and stored at −20 °C. Real-time PCR was performed using sense and antisense primers. The primer pairs were as follows: mdr1a, sense 5′-GGATGAAATTGATAATTTAGACATG-3′ and antisense 5′-TCCATTTATTATGGCACAGAATATA-3′; β-actin, sense 5′-TCT GTG TGGA TTG GTG GCT CTA-3′ and antisense 5′-CTG CTT GCT GAT CCA CAT CTG-3′. PCR amplification was performed using SYBR Green PCR reagents and the ABI PRISM 7300 sequence detection system (Applied Biosystems, Foster City, CA). The relative amount of target mRNA was determined using the comparative threshold (Ct) method by normalizing target mRNA Ct values to those for β-actin (ΔCt). Statistical analysis of real-time PCR data was performed using ΔCt values.

### Western blot

For protein extraction, the cells or mice liver tissues were homogenized in RIPA buffer and then centrifuged at 14,000 g at 4 °C for 30 min. The supernatant was collected and the protein concentration was measured using BCA assay. The sample (50 μg protein/sample) was then loaded on 12% polyacrylamide gels and transferred to polyvinylidene difluoride membranes (Millipore, USA). After blocked by 3% bovine serum albumin, the membranes were incubated with primary antibodies and HRP-conjugated secondary antibody, and then detected using the enhanced chemiluminescence plus detection system (Molecular Device, Lmax, USA). The density of each band was quantified by using image analysis software (Science Lab 2005 Image Guage; Tokyo, Japan).

### Toxicokinetic study

For TP plasma kinetic study and toxicological evaluation, mice were divided into four groups (n = 5 each) to collect blood and tissue samples: (1) normal + saline group; (2) 1.0 mg/kg TP + 15 nmol NC-siRNA group; (3) 1.0 mg/kg TP + 15 nmol mdr1a-siRNA group; (4) 1.0 mg/kg TP + 10 mg/kg tariquidar group. In order to avoid the complication caused by drug absorption or possible intestinal first-pass effect, TP and the inhibitor were intravenously administrated to mice. The siRNA group was intravenously injected with NC-siRNA or mdr1a-siRNA 2 days before TP dose. For TP + tariquidar group, the mice were received an intravenous tariquidar dose 20 min prior to the TP injection. Blood samples were collected at 2, 5, 10, 15, 30, 60 and 120 min after TP dosing. To assess the liver exposure of TP, liver tissue samples were collected from another set of mice at 5, 30, 60 and 120 min after dosing. Three TP groups were design for this experiment, including TP + NC-siRNA group, TP + mdr1a-siRNA group and TP + tariquidar group. The liver tissue samples were weighed and then homogenized in 10 volume (w:v) of ice-cold saline. The concentrations of TP in plasma and liver tissue were measured by a validated LC-MS/MS method. Prior to the above animal study, a preliminary experiment was performed to assess the possible differences in liver P-gp protein expression (Figure S1) and TP plasma kinetics between NC-siRNA treated mice and normal mice (with saline) (Figure S2 and supplemental Table 1). No significant differences were observed in the TP plasma concentrations and kinetic parameters. Therefore, NC-siRNA group was used as the control in the following animal study.

### Toxicological evaluation

At 24 h of TP treatment, blood and liver tissue samples were collected from the mice in plasma kinetic study. Serum ALT and AST levels were determined with a CL8000 automated biochemical analyzer (Shimadzu, Japan) after serum was separated by centrifugation at 900 g for 10 min. Liver tissue samples were divided into two parts, the first half was kept at −80 °C until MDA and SOD measurements using MDA and SOD assay kits (Nanjing Jiancheng Bioengineering Institute, Nanjing, China) according to the manufacturer’s instructions. The other half was fixed with 4% paraformaldehyde solution and then dehydrated, embedded in paraffin and sectioned at a thickness of 5 μm. Four paraffin sections from each mouse were dewaxed, rehydrated and stained with hematoxylin-eosin (HE) for overall morphological evaluation. The total protein concentration was quantified by the BCA assay. Bcl-2 and Bax proteins were measured by western blot.

### Quantification of TP

The TP in plasma and tissue samples was quantitatively analyzed by a published LC-MS/MS method^18^. The method was slightly modified and validated for analysis of plasma and liver tissue samples. Plasma and tissue homogenate were pretreated with 3-fold volume of methanol-acetonitrile (1:1, v/v) containing 20 ng/ml propranolol to precipitate proteins, and then centrifuged at 4°C, 14,000 g for 10 min. The supernatant (10 μl) was injected into LC-MS/MS for analysis. An Aglient 6410B triple quadrupole mass spectrometer equipped with Agilent1290 Infinity UHPLC system (Agilent Technologies, Santa Clara, CA) was used. TP and propranolol (internal standard, IS) were eluted from an Agilent C18 column (dp = 3.5 μm, 2.1 × 50 mm, Agilent, MA) using a mobile phase gradient (A: water with 0.1% formic acid and 2.5 mM ammonium formate, B: acetonitrile with 0.1% formic acid). The gradient was run as: 0–2.5 min hold at 95% B; 2.5–3 min, a linear gradient to 10% B; 3–4 min hold at 10% B. Flow rate was 0.2 ml/min. TP and propranolol were detected in positive ion mode using single reaction monitoring: TP, m/z 378.1/360.1, IS, m/z 260.0/116.0. Under the above optimized condition, no significant endogenous interferences were detected at the peak regions for TP and IS in all the biological specimens. The calibration curves of TP were linear (r^2^ > 0.99) over the concentration range of 1–1000 ng/ml. The lower limit of quantification was at 1 ng/ml. The intra and inter-day precisions (RSD) were all below 11% and the assay accuracies (RE) were in the range from 87% to 113%. The recovery was above 80% for both plasma and tissue samples.

### Molecular docking

To compare the binding modes of TP and tariquidar on P-gp, a molecular docking analysis was performed. The crystal structure of mouse P-gp (PDB: 3G5U) was selected as the receptor for molecular analysis, as mouse was the animal model used in the present study and the mouse P-gp bears a high degree of homology (about 87% amino acid identity) with human. The initial ligand molecule structures (TP and tariquidar) were retrieved from the NCBI-PubChem Compound Database. The docking simulation was performed with AutoDock 4.2, a molecular modeling simulation program that is widely used for the automated docking of ligands and their macromolecular receptors. The free energies of binding for the protein/modulator complexes were estimated from the equilibrated trajectories using the SIETRAJ algorithm^33^. The conformation corresponding to the lowest energy was selected as the most probable binding conformation^32^.

### Data analysis

Toxicokinetic parameters, including area under the plasma concentration-time curve (AUC), volume of distribution (Vd), clearance (CL), mean residence time (MRT) and terminal elimination half-life (t_1/2_), were calculated by non-compartmental analysis using WinNonlin 5.2 software (Pharsight Corporation, CA, USA). All the data were expressed as mean ± SD. Differences between groups were compared by Student’s t-test for analysis of unpaired data or one-way ANOVA. Statistical significance was accepted at *P* < 0.05.

## Results

### Screening and functional evaluation of mdr1a siRNA

**Figure 2:**
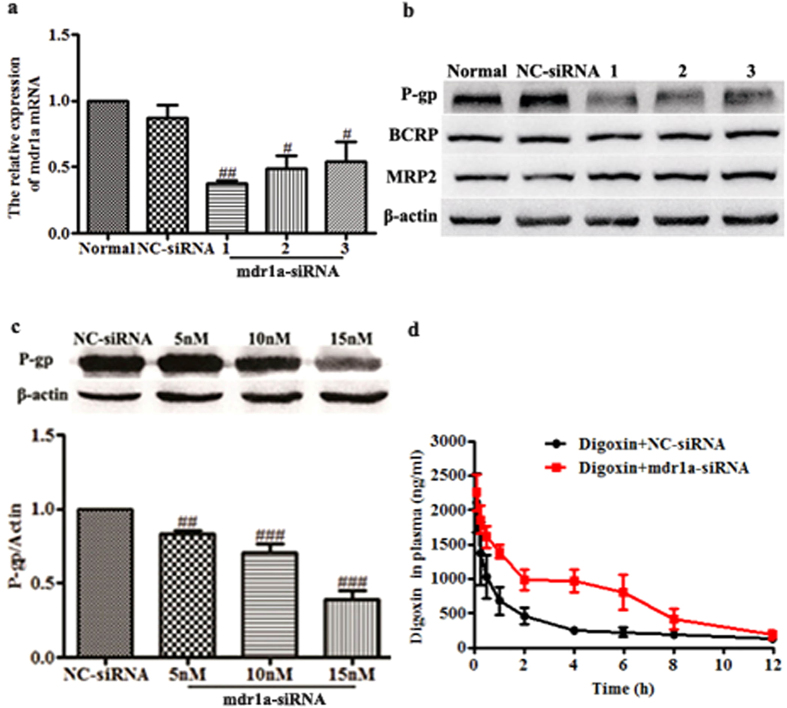
Screening and functional evaluation of mdr1a siRNA *in vitro and in vivo*. (**a**) RNAi-mediated knockdown of mdr1a mRNA in CT26.WT cells. (**b**) RNAi-mediated knockdown of P-gp protein in CT26.WT cells. CT26.WT cells were transfected with mdr1a siRNAs. After 48 h, mRNA for mdr1a gene and P-gp protein expression were measured by real-time PCR and western blot. (**c**) RNAi-mediated knockdown of P-gp protein expression in liver of mice. mdr1a-1 siRNA (at a dose of 5, 10, 15 nmol) was injected via the tail vein of mice. After 48 h, the livers of mice were collected and the P-gp protein expression was measured to validate the efficacy of siRNA. (**d**) Evaluation of P-gp function after pretreatment with mdr1a-1 siRNA in mice. The NC-siRNA or mdr1a-1 siRNA (15 nM) was intravenously injected 2 days prior to the digoxin injection at a dose of 1 mg/kg. The blood samples were collected at scheduled time intervals. Data are presented as mean ± SD (n = 3 *in vitro* and n = 5 *in vivo*). ^#^*P* < 0.05, ^##^*P* < 0.01, ^###^*P* < 0.001 compared with NC-siRNA group.

In this study, CT26.WT cells were transfected with various siRNA sequences against mdr1a. The suppressive effect was measured at 48 h after transfection to evaluate the specificity of siRNA targeted to mdr1a. Figure 2a showed the silencing efficiency of three siRNA sequences on the same mRNA target. All the three siRNA sequences inhibited the expression of mdr1a mRNA. However significant differences in silencing efficiency were observed, with 63%, 51% and 46% for mdr1a-1, mdr1a-2 and mdr1a-3 siRNA, respectively. Western blot assay further indicated that the P-gp expression in the cells treated with these mdr1a siRNA was also remarkably reduced (Fig. 2b). Among the three sequences, mdr1a-1 was the most efficient one for reducing both the expression of mdr1a mRNA and protein. In addition, the protein expressions of BCRP and MRP2 were not changed, which confirmed the specificity of mdr1a-1 siRNA (Fig. 2b). Therefore, mdr1a-1 siRNA was selected for the *in vivo* study.

The suppressive effect on P-gp expression in mouse liver was determined by western blot at 48 h after the mice received an intravenous injection of mdr1a-1 siRNA. Figure 2c showed that, when compared to the control group, the expression of P-gp protein in liver of the mdr1a-siRNA group was significantly reduced. The silencing efficiency was calculated to be 62%, which was consistent with the *in vitro* reduction at the mRNA level.

Using the known P-gp substrate digoxin, the *in vivo* suppressive effect of the siRNA on P-gp function was further assessed. The plasma concentration-time profile of digoxin in mice after P-gp knockdown was shown in Fig. 2d, and the pharmacokinetic parameters were listed in Table 1. In the pretreatment group with mdr1a-1 siRNA, the AUC_0-t_ of digoxin was increased to about 2.6-fold of that for NC-siRNA group. Meanwhile, Vd and CL of digoxin were decreased to 39% and 30% of NC-siRNA group, respectively. The results indicated that the selected mdr1a-1 siRNA was specific and effective for down-regulation of P-gp function in mice.

### The effects of siRNA and tariquidar on TP plasma and hepatic exposures

**Figure 3:**
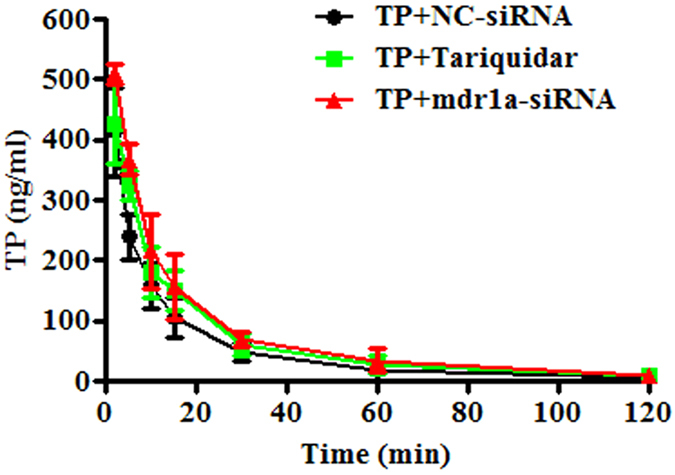
Effects of mdr1a-siRNA and tariquidar on triptolide toxicokinetics in mice. The NC-siRNA or mdr1a-siRNA (15 nM) was intravenously injected 2 days and tariquidar (10 mg/kg) was intravenously given 20 min prior to the injection of triptolide at a dose of 1 mg/kg. The blood samples were collected at scheduled time points. Data are presented as mean ± SD (n = 5).

Toxicokinetic profiles of TP for control (TP+NC-siRNA), TP + mdr1a-siRNA and TP + tariquidar groups were showed in Fig. 3. The toxicokinetic parameters were calculated and listed in Table 2. The TP plasma concentrations were declined rapidly in mice after received an intravenous dose. After 2h of injection, the TP concentrations were dropped below the lower limit of quantification for all three groups. A comparison of the parameters was made between the control and the treated groups to assess the effect of P-gp inhibition on the TP exposure and elimination. Treatment with the mdr1a-siRNA could significantly enhance the TP plasma exposure, with the C_max_ increased from 413 ± 74 to 510 ± 94 ng/ml (*P* < 0.05) and the AUC from 103.5 ± 9.6 to 154.3 ± 30.2 ng·h/ml (*P* < 0.05). In the concomitant group with tariquidar, the significantly increased AUC was also noted, from 103.5 ± 9.6 of the control to 145.9 ± 24.6 ng·h/ml of the TP+tariquidar group (*P* < 0.05). Accordingly, the total body clearance of TP in mice was remarkably decreased, from 9564.0 ± 1024.2 ml/min/kg of the control to 6576.4 ± 1438.5 (*P* < 0.05) and 5755.4 ± 1200.1 ml/min/kg (*P* < 0.05) for TP+tariquidar and TP+mdr1a-siRNA groups, respectively. Although not significant, the t_1/2_ and MRT of the two treatment groups showed a tendency of increases, in agreement with the decreased clearance parameters for the same groups.

TP concentrations in the liver tissues were also determined to link the hepatic exposure with hepatotoxicity. As shown in Table 3, pretreatment with the mdr1a-siRNA markedly increased the TP hepatic exposure and the levels were up to 2.2, 2.4, 1.9 and 2.3-fold of the control group at 5, 30, 60 and 120 min, respectively. Similarly, the TP exposure in liver was also enhanced by tariquidar, with the concentrations about 1.5-fold of the control. The results suggested that suppression of TP canalicular efflux by P-gp gene knockdown or chemical inhibition could significantly increase its systemic and hepatic exposures. This might lead to severe hepatotoxicity.

### The effects of siRNA and tariquidar on TP-induced hepatotoxicity in mice

**Figure 4:**
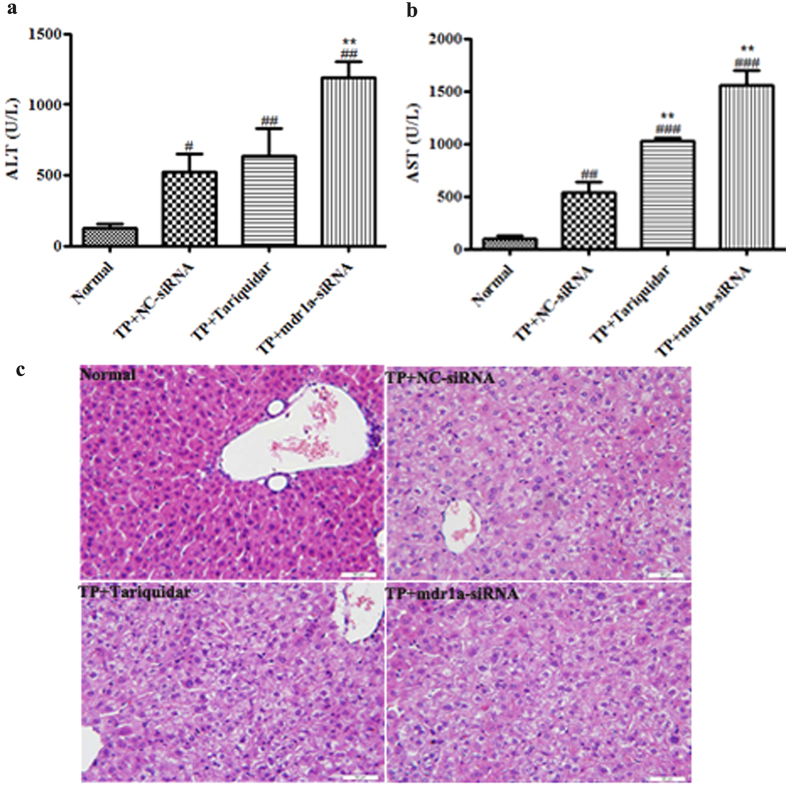
Effects of mdr1a-siRNA and tariquidar on triptolide induced releases of serum biochemical markers and pathological changes in mice. The NC-siRNA or mdr1a-siRNA (15 nM) was intravenously injected 2 days and tariquidar (10 mg/kg) was intravenously given 20 min prior to the injection of triptolide at a dose of 1 mg/kg. Blood samples were collected for the measurement of ALT and AST at 24 h after dosing. Livers were collected for pathological examination at 24 h after dosing. Sections were H&E-stained and Scale bar = 50 μm. Data are presented as mean ± SD (n = 5). ^#^*P* < 0.05, ^##^*P* < 0.01, ^###^*P* < 0.001 compared with normal group, ^**^P < 0.01 compared with NC-siRNA group. (**a**) the level of ALT; (**b**) the level of AST; (**c**) H&E-staining.

To determine the impact of impaired P-gp function on TP-induced toxicity, serum biochemical measurement and histopathological examination were carried out at 24h of TP treatment. The serum ALT and AST levels are commonly used as the biochemical markers for liver injury. In the TP+NC-siRNA group, TP lifted the ALT and AST levels up to 4.2 and 5.4 folds of the normal group, indicating the TP-induced toxicity (Fig. 4a,b). Down regulation of P-gp by pretreatment of mdr1a-siRNA could further remarkably increase the serum levels of ALT and AST up to 2.3 and 2.9 folds of the TP+NC-siRNA group, respectively. In the TP+ tariquidar group, the serum levels of ALT and AST were 1.2 and 1.9 folds of the TP+NC-siRNA group. The results clearly demonstrated the severe liver injury in the mice with P-gp gene knock down or functional inhibition.

Histopathological examination of the livers was performed to check the liver injury caused by TP and the additional treatment (Fig. 4c). When compared to the normal histology of the hepatocytes, the liver sections from the mice in the NC-siRNA group showed hepatocellular hydropic degeneration and vacuolization and these pathological changes were more severe in the tariquidar concomitant group. In the mdr1a-1 siRNA pretreatment group, in addition to the above observed pathological changes, eosinophilic necrosis of the hepatocytes was also noted. The results were consistent with the enhanced hepatic exposure of TP as well as serum levels of ALT and AST.

### Alteration in biomarkers of oxidative stress and apoptosis-related proteins

**Figure 5:**
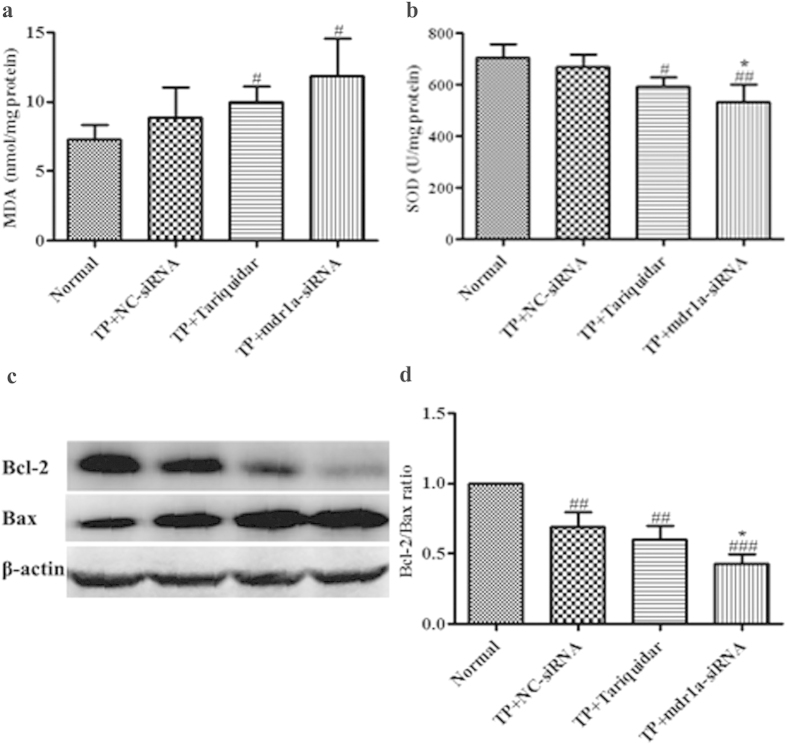
Effects of mdr1a-siRNA and tariquidar on the content of MDA, the activities of SOD and the expression of Bcl-2 and Bax in the liver of triptolide treated mice. The NC-siRNA or mdr1a-siRNA (15 nM) was intravenously injected 2 days and tariquidar (10 mg/kg) was intravenously given 20 min prior to the injection of triptolide at a dose of 1 mg/kg. Livers were collected at 24 h after dosing. Data are presented as mean ± SD (n = 5). ^#^*P* < 0.05, ^##^*P* < 0.01, ^###^*P* < 0.001 compared with normal group, ^*^P < 0.05 compared with NC-siRNA group. (**a**) the content of MDA; (**b**) the activities of SOD; (**c**) the protein bands of Bcl-2 and Bax; (**d**) quantitative analysis of Bcl-2/Bax ratio.

The early studies suggested that the excessive apoptosis of hepatocytes, the lipid peroxidation and the inhibition of mitochondrial respiratory chain were the possible mechanism of TP-induced hepatotoxicity^34^,^35^,^36^. To explore the relation of the TP hepatic exposure with these toxicological pathways, we further measured the MDA and SOD levels and the expression of Bcl-2 and Bax proteins in the mice livers. MDA and SOD are the indicators of oxidative stress^37^. The results demonstrated that the hepatic MDA level was increased in all three TP treatment groups at 24h of TP dosing (Fig. 5a). However, the statistically significant increase (*P* < 0.05) was observed only for TP+tariquidar group (1.3 fold) and TP+mdr1a-siRNA group (1.6 fold). The similar pattern was seen in the SOD activity. Although TP induced a reduction of SOD activity in all three TP groups (Fig. 5b), the statistically significant differences were only noted in the groups treated with tariquidar (*P* < 0.05) and mdr1a-siRNA (*P* < 0.01). The increase in MDA level and the reduction in SOD activity were proportional to the TP exposure in liver, suggesting that the mice with the down-regulated P-gp function were more sensitive to the TP-induced toxicity.

The Bcl-2 family proteins are key regulators of apoptosis, which include both anti-apoptotic members such as Bcl-2 and the pro-apoptotic members such as Bax. A slight change in the dynamic balance of these proteins may result either in inhibition or promotion of cell death^38^,^39^. In this study, the expression levels of Bcl-2 and Bax were measured with western blot to assess the degree of the TP induced apoptosis. The results in Fig. 5c,d showed the significantly reduced Bcl-2/Bax ratios in three TP treated groups, as the result of the markedly decreased Bcl-2 expression level and the increased Bax level. In consistent with the changes in TP hepatic exposure, the highest rate of apoptosis was shown in TP+mdr1a-siRNA group, which had the lowest Bcl-2/Bax ratio (Fig. 5d) compared to the normal (*P* < 0.001) and TP + NC-siRNA group (*P* < 0.05), respectively. Concomitant with tariquidar also markedly reduced the ratio when compared to the normal group (*P* < 0.01). The reduction of Bcl-2/Bax ratio was negatively correlated to the hepatic exposure of TP (*r*^2^ = 0.95), indicating that the TP induced cell apoptosis was associated with its hepatic exposure and aggravated by the impaired P-gp function.

### Molecular docking of TP and tariquidar binding on P-gp protein

**Figure 6:**
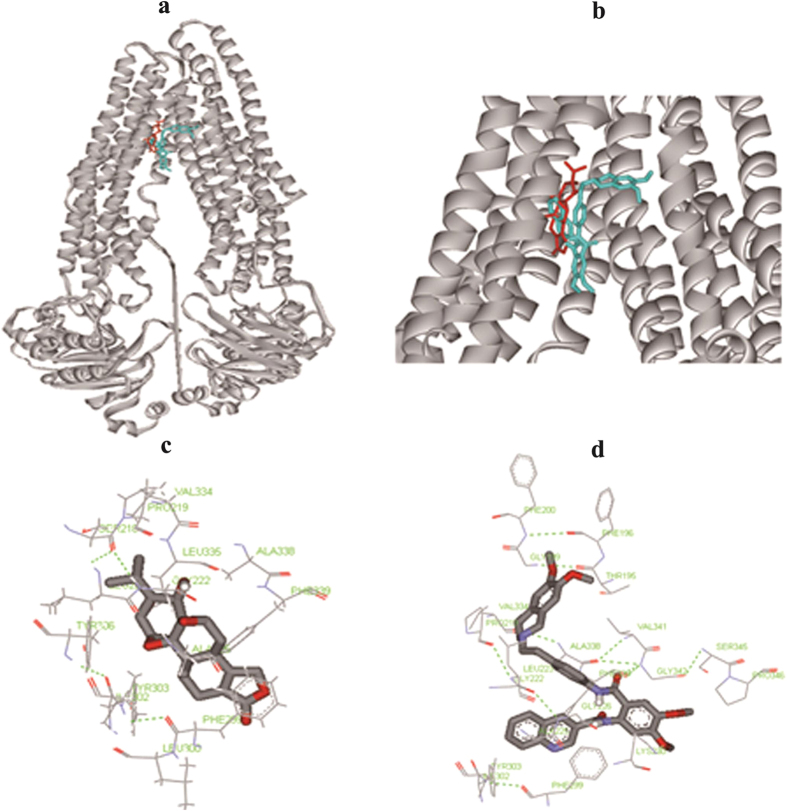
Molecular docking of triptolide and tariquidar to mouse P-gp. The conformation corresponding to the lowest energy was selected as the most probable binding positions. (**a**) The three-dimensional structure of the binding site cavity of P-gp bound to tariquidar (cyan), triptolide (red). (**b**) Magnified view of the binding site cavity shown in (A). (**c**) Stereo view of triptolide-interacting residues within the active site region of P-gp. (**d**) Stereo view of tariquidar-interacting residues within the active site region of P-gp.

The above results indicated that tariquidar, a known P-gp inhibitor, could remarkably increase the plasma and liver exposures of TP by inhibiting its efflux transport. To further understand the binding property of TP with P-gp protein, a molecular docking analysis was performed at the reported tariquidar-binding sites on P-gp^33^. The molecular binding modes were also compared between TP and tariquidar (Fig. 6a,b). The hydrophobic cavity could be observed in the binding site for both TP and tariquidar. In the internal cavity of P-gp, a number of amino acid residues were responsible for the binding of tariquidar and TP (Fig. 6c,d). Among these residues, Phe299, Phe339, Ile302, Ala225, Ala338, Pro219, Val334 and Tyr303 were common for TP and tariquidar in hydrophobic cavity of P-gp, which suggested that tariquidar and TP shared some binding sites on P-gp protein. The lowest binding energies of the protein/compounds complexes were −10.1 and −8.24 KJ/mol for tariquidar and TP, respectively. Lower binding energy of tariquidar over TP explained its inhibitory effect on P-gp mediated TP transport.

## Discussion

As the major active constituent of TWHF, TP has been proved to be very effective in treatment of rheumatoid arthritis and system lupus erythematosus^40^. However, the TP-induced liver toxicity has recently caused increasing safety concerns in clinic^7^,^8^. Numerous investigations were conducted to explore the relation between the drug disposition and toxicity, with the focus mostly on the CYP metabolism. The parent form of TP was confirmed to be responsible for the toxicity and the CYP3A mediated metabolism was a detoxification pathway. In addition to the CYP3A mediated clearance, our previous study revealed that efflux transporter mediated hepatobiliary excretion was also an important clearance route for TP and the canalicular P-gp exerted a significant role in TP induced hepatotoxicity in SCRH^18^. In the present study, we further presented the *in vivo* evidence that P-gp contributed significantly to TP clearance and detoxification. The significantly increased TP exposure in liver and plasma in the P-gp down-regulated mice was clearly correlated to the enhanced hepatotoxicity.

In this study, RNAi technique was selected as the primal tool to assess the contribution of P-gp protein to the TP clearance, as TP was a dual substrate of CYP3A and P-gp. The silencing efficiency of 62% was obtained in this study for the synthetic siRNA at liver protein level of murine mdr1a in mice. It was slightly higher than the efficiency (50–60%) reported by Matsui’s group^30^. Using a known P-gp substrate digoxin, the down-regulated P-gp function by the mdr1a-siRNA was further confirmed with 60% reduction noted in the total body clearance (CL) of the substrate. In this P-gp knockdown model, the contribution of P-gp in TP clearance and detoxification was clearly demonstrated. Comparing to the TP+NC-siRNA group, the increase of 1.5-fold in the plasma AUC and of 1.9 to 2.4-fold in liver exposure at different time points was observed for the TP+mdr1a-siRNA group (Table 3). The increase fold of TP hepatic exposure in P-gp knockdown mice (2.3 fold at 120 min) was comparable to that in CYP KO mice (about 2-fold at 120 min) reported by Xue and coworkers using CYP reductase KO mice^11^. As the result of higher exposure of TP in liver, the aggravated hepatotoxicity was observed. A 2.9-fold of increase in the AST level was determined in the P-gp knockdown mice in the present study and a nearly 2-fold increase was seen in CYP KO mice in Xue’s study^11^. The above observations indicated that the P-gp mediated clearance played an important role in the detoxification of TP *in vivo*, with the contribution comparable to the CYP mediated metabolism. This was also in agreement with our *in vitro* result that CYP3A and P-gp likely contributed equally to the detoxification of TP in sandwich-cultured rat hepatocytes model^18^.

Drug interactions usually occur when one drug alters the pharmacokinetics of another drug by influencing its absorption, distribution and clearance. In assessment of the pharmacokinetics mediated DDI, a common approach is concomitant use of the substrate drug with inhibitors or inducers of drug metabolizing enzymes or drug transporter proteins^19^,^24^,^41^. In this study, tariquidar was selected to assess the potential DDI with TP. The previous studies have demonstrated that tariquidar was a potent and selective inhibitor of P-gp protein with much less enzyme based pharmacokinetic interaction than earlier used P-gp inhibitors, such as cyclosporin A and ritonavir^42^,^43^. It could fully restore the antitumor activity of cytotoxic drug at a dose of 10 mg/kg, without displaying CYP mediated pharmacokinetic interactions^44^. In our study, concomitant with tariquidar (10 mg/kg) significantly altered both systemic and hepatic exposures of TP. The plasma AUC was lifted to 1.4-fold and CL decreased to 69% of the control group. Subsequently, the aggravated liver injury was observed with the significantly increased ALT and AST levels and pathological changes. To get the insights of the tariquidar inhibition on TP efflux and the molecular interaction between TP, tariquidar and P-gp protein, an in silico method was used to compare the binding property of TP and tariquidar on P-gp protein. Some published studies have demonstrated the success of using molecular docking technique to predict features of compounds for P-gp recognition and to model the interaction of substrates and modulators with P-gp^32^,^45^. In our study, molecular docking analysis of TP and tariquidar was performed with AutoDock, a software system widely used to predict the optimal binding conformations of ligands to proteins^46^,^47^. The result indicated that TP shared the same binding pocket with tariquidar (Fig. 6). They both bound to a number of amino acid residues, such as Phe299, Phe339 and Val334. And lower binding energy of tariquidar indicated that it could compete with TP on the P-gp binding sites and led to the efflux transporter mediated DDI. Although concomitant with tariquidar only cause 1.4-fold increase in plasma AUC, the toxicological consequence was more severe due to the narrow therapeutic window of TP. The potential risk of herb-drug interaction or herb-herb interaction likely occurs when TP is used in combination with P-gp inhibitors, such as tariquidar.

In this study, knockdown of P-gp by mdr1a-siRNA showed a higher effectiveness over tariquidar. Specific siRNA designed to target mdr1a or mdr1a/1b gene have been showed an excellent potential in down-regulation of P-gp expression and function *in vitro* and *in vivo*^48^,^49^,^50^. This is owed to high specificity and efficiency of siRNA and its inhibitory effect on gene expression at transcription level, which is usually more powerful than the inhibition at functional level. Tariquidar is a chemical inhibitor of P-gp and its modulating effect is derived from the competition with substrates at P-gp binding sites, inhibition of ATP hydrolysis or both^51^. The effect of tariquidar on canalicular P-gp is also relied on the exposure level at its targeted tissue/organ, which may be affected by the dose level, dosing route and also disposition fate *in vivo*. Therefore, siRNA is an appropriate technique to assess the role of transporters in disposition and toxicity of drugs or xenobiotics *in vivo*.

**Figure 7:**
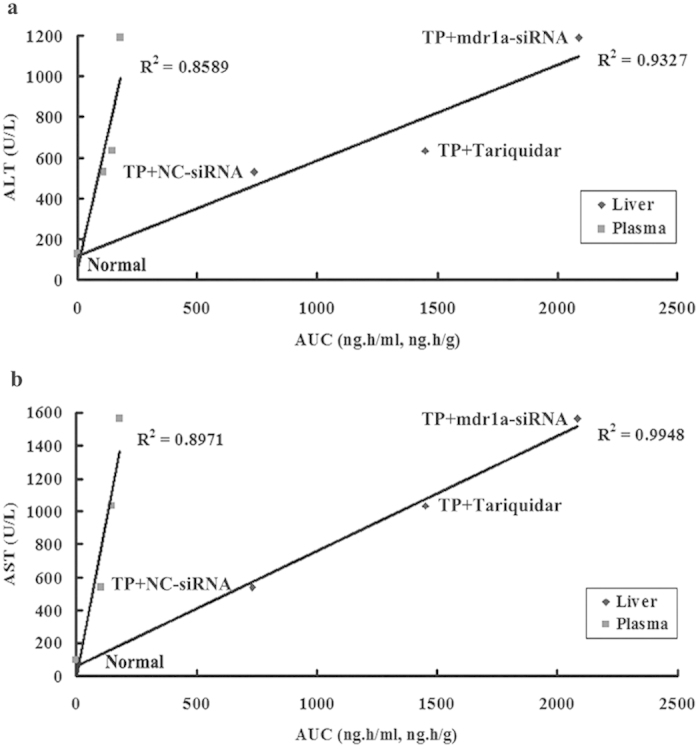
Correlation of triptolide-induced hepatotoxicity with its systemic and hepatic exposures. Mice were treated with or without triptolide (1 mg/kg) in the presence of siRNA and tariquidar. (**a**) the correlation of ALT with systemic and hepatic exposures; (**b**) the correlation of AST with systemic and hepatic exposures.

To analyze the relation between TP exposures and hepatotoxicity, we plotted serum ALT or AST level against TP exposure (AUC) for the TP treatment groups with or without modulators. As illustrated in Fig. 7, good quantitative correlations were observed between marker release (AST or ALT) and plasma (*r*^2^ of 0.8971 for AST and 0.8589 for ALT) or hepatic exposures (*r*^2^ of 0.9948 for AST and 0.9327 for ALT). Pretreatment with mdr1a-siRNA resulted in the highest TP exposure in plasma and liver, corresponding to more severe toxicity, with about 60% of the death rate within 30 h of TP treatment. The Fig. 7 also showed that the slope of plasma plot was much sharper (5.62 for ALT and 7.68 for AST) than that of the liver (0.47 for ALT and 0.70 for AST), indicating that a small change in plasma concentration corresponded to a high degree of liver damage. The narrow therapeutic window of TP in term of plasma concentration would make it difficult in clinical practice to predict TP induced hepatotoxicity through plasma concentration monitoring. Instead, serum biochemical markers would be more appropriate indicators for use in clinic to monitor the possible DDI mediated alteration in the hepatic TP exposure and subsequently the hepatotoxicity.

Apart from the serum biomarkers, more toxicological evaluations were performed based on the known mechanism of TP toxicity. The levels of MDA and SOD, together with the expression of Bax and Bcl-2 proteins in the liver were measured to assess the impact of P-gp modulation on the TP-induced toxicity. The data showed in Fig. 5 clearly indicated that down-regulation of P-gp could significantly enhance TP-induced oxidative stress and cell apoptosis. The severity of the toxicological effects was correlated to the hepatic exposure of TP. The TP toxicity was largely relied on its hepatic clearance. Any factor that affects the metabolism or biliary excretion of TP might cause safety risk in clinic. Besides the modulation of metabolizing enzymes and efflux transporters by drug-drug and drug-herb interaction, some physiological and pathological condition may also have impacts on the TP-induced hepatotoxicity, for instance, hepatic impairment and bile duct blockage.

In summary, our results demonstrated that knockdown of hepatic P-gp significantly altered the systemic and hepatic exposures of TP *in vivo*, leading to severe hepatotoxicity. The hepatotoxicity was more relevant to the hepatic TP exposure. P-gp mediated clearance contributed significantly to the detoxification of TP. In clinical practice, the potential risk of herb-drug interaction or herb-herb interaction must be closely monitored when TP is concomitant with P-gp inhibitors or substrates.

## Additional Information

**How to cite this article**: Kong, L.-L. *et al.* Inhibition of P-glycoprotein Gene Expression and Function Enhances Triptolide-induced Hepatotoxicity in Mice. *Sci. Rep.* **5**, 11747; doi: 10.1038/srep11747 (2015).

## Supplementary Material

Supplementary Information

## Figures and Tables

**Table 1:** Pharmacokinetic parameters of digoxin in mice.

Parameters	Digoxin+NC-siRNA	Digoxin+mdr1a-siRNA
t_1/2_ (h)	5.7 ± 1.3	4.0 ± 1.4
C_max_ (ng/ml)	1737.8 ± 573.7	2255.4 ± 266.9
AUC_(0-t)_ (ng·h/ml)	3363.3 ± 739.1	8774.0 ± 1448.7^##^
CL (ml/min/kg)	252.8 ± 62.9	99.8 ± 22.7^##^
Vd (L/kg)	2.0 ± 0.5	0.6 ± 0.08^###^
MRT (h)	3.7 ± 0.3	3.8 ± 0.4

Data are presented as mean ± SD (n = 5). ^##^*P* < 0.01, ^###^*P* < 0.001, compared with NC-siRNA group.

**Table 2:** Toxicokinetic parameters of triptolide in mice.

Parameters	TP+NC-siRNA	TP+Tariquidar	TP+mdr1a-siRNA
t_1/2_ (h)	0.34 ± 0.14	0.49 ± 0.22	0.49 ± 0.23
C_max_ (ng/ml)	413.1 ± 74.2	471.6 ± 66.5	509.7 ± 94.1^#^
AUC_(0-t)_ (ng·h/ml)	103.5 ± 9.6	145.9 ± 24.6^#^	154.3 ± 30.2^#^
CL (ml/min/kg)	9564.0 ± 1024.2	6576.4 ± 1438.5^#^	5755.4 ± 1200.1^#^
V_d_ (L/kg)	4.8 ± 1.1	3.9 ± 1.3	3.3 ± 0.7^#^
MRT (h)	0.34 ± 0.13	0.50 ± 0.22	0.51 ± 0.23

Data are presented as mean ± SD (n = 5). ^#^*P* < 0.05, compared with NC-siRNA group.

**Table 3:** Hepatic concentrations of triptolide in mice.

TP (ng/g protein)	5 min	30 min	60 min	120 min
TP+NC-siRNA	1953.3 ± 786.7	394.9 ± 41.2	170.6 ± 66.3	37.4 ± 12.2
TP+Tariquidar	2857.3 ± 909.4	683.9 ± 61.3^#^	302.8 ± 22.7^#^	36.1 ± 14.4
TP+mdr1a-siRNA	4278.0 ± 653.1^#^	950.8 ± 232.1^#^	326.2 ± 22.4^#^	84.8 ± 14.5^#^

Data are presented as mean ± SD (n = 5). ^#^*P* < 0.05, compared with NC-siRNA group.
