# Supplementary material for: Inhibition of P-glycoprotein Gene Expression and Function Enhances Triptolide-induced Hepatotoxicity in Mice
Source: Sci Rep. 2015 Jul 2;5:11747. doi: 10.1038/srep11747 (PMC4488747; doi:10.1038/srep11747)
Supplement: Supplementary Information [file srep11747-s1.pdf]

Supplemental information

Inhibition of P-glycoprotein Gene Expression and Function Enhances Triptolide-induced Hepatotoxicity in Mice

Ling-Lei Kong[1,2], Xiao-Mei zhuang[1,2], Hai-Ying Yang[1,2], Mei Yuan[1,2], Liang Xu[1,2], Hua Li[1,2,*],

[1]State key laboratory of Toxicology and Medical Countermeasures, Beijing 100850, China.

[2]Beijing Institute of Pharmacology and Toxicology, Beijing 100850, China.

[*]**Corresponding author:**

Hua Li,

Beijing Institute of Pharmacology and Toxicology, 27 Taiping Road, Haidian District, Beijing, 100850, China.

Tel: +86 10 66930664, Fax: +86 10 68211656, E-mail: amms_lee@163.com.

Experimental Section

Effect of NC-siRNA on P-gp protein expression *in vivo*

For evaluation of NC-siRNA on P-gp protein expression in vivo, NC-siRNA was injected via the tail vein of mice after diluted to 5, 10 and 15 nmol with saline in 200 μl. After 48 h, the mice were euthanized and the liver was collected for measurement of the P-gp protein expression using western blot.

Effect of NC-siRNA on pharmacokinetic of TP

Mice were divided into two groups to collect blood samples (n=5 each): (1) 1.0 mg/kg TP + saline group; (2) 1.0 mg/kg TP + 15 nmol NC-siRNA group. The siRNA group was intravenously injected with NC-siRNA 2 days before TP dose. Blood samples were collected at 2, 5, 10, 15, 30, 60 and 120 min after TP dosing. The concentrations of TP in plasma and liver were measured by a validated LC-MS/MS method.

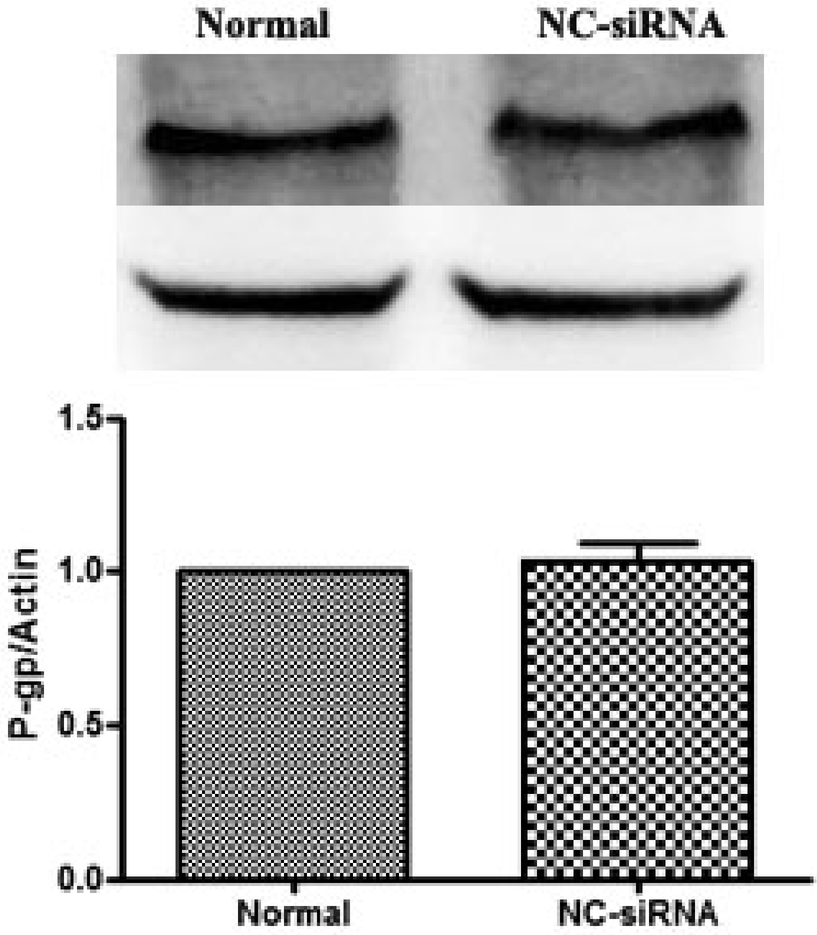

Figure S1 Effect of NC-siRNA on P-gp protein expression in liver of C57 mice

The mice received an injection dose of NC-siRNA (15 nM) via the tail vein. After 48 h, the livers of mice were collected and the P-gp protein expression was measured. Data are presented as mean ± SD (n=5).

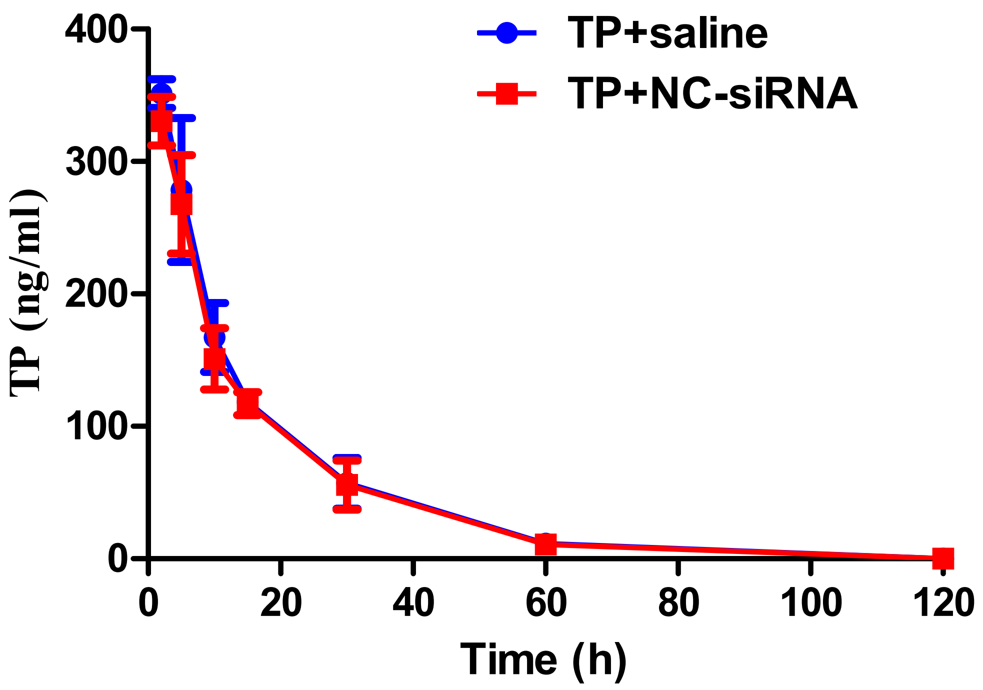

Figure S2 Effect of NC-siRNA on toxicokinetics of triptolide in mice.

The NC-siRNA (15 nM) was intravenously injected 2 days prior to the injection of triptolide at a dose of 1mg/kg. The blood samples were collected at scheduled time intervals. Data are presented as mean ± SD (n=5).

Supplementary Table 1 Pharmacokinetic parameters of TP in mice.

Parameters	TP+saline	TP+NC-siRNA
$t_{1/2}$ (h)	0.21±0.05	0.21±0.06
C_{max} (ng/ml)	351.3±10.9	330.5±18.2
$AUC_{(0-t)}$ (ng·h/ml)	103.8±13.4	99.1±11.7
CL (ml/min/kg)	9914.6±1254.4	10352.2±1157.9
V_d (L/kg)	3.0±0.3	3.1±0.6
MRT (h)	0.23±0.03	0.26±0.04

Data are presented as mean ± SD (n=5)
